# Comment on Aaseth et al. Circulating Lipoproteins in Subjects with Morbid Obesity Undergoing Bariatric Surgery with Gastric Bypass or Sleeve Gastrectomy. *Nutrients* 2022, *14*, 2381

**DOI:** 10.3390/nu15010041

**Published:** 2022-12-22

**Authors:** Sílvia Paredes, Jonatas Barbosa Garcez, Laura Ribeiro

**Affiliations:** 1 Endocrinology Department, Centro Hospitalar Universitário do Porto, Largo do Prof. Abel Salazar, 4099-001 Porto, Portugal; 2 Pathology Department, Centro Hospitalar Universitário do Porto, Largo do Prof. Abel Salazar, 4099-001 Porto, Portugal; 3 Medical Education Unit, Department of Public Health and Forensic Sciences, and Medical Education, Faculty of Medicine, University of Porto, 4200-319 Porto, Portugal; 4 I3S-Instituto de Investigação e Inovação em Saúde, Universidade do Porto, 4200-135 Porto, Portugal

We read with great interest the paper by Jan O. Aaseth et al. entitled “Circulating Lipoproteins in Subjects with Morbid Obesity Undergoing Bariatric Surgery with Gastric Bypass or Sleeve Gastrectomy” where changes in lipid profile after bariatric surgery were reported [1].

Despite astonishing advances in the treatment of atherosclerotic cardiovascular disease (ACVD), it remains the leading cause of death in developed countries. Thus, reporting variation in major lipoproteins after bariatric surgery is of great scientific interest. The cited paper was particularly relevant for not only evaluating the traditional components of lipid profile but other atherogenic variables, such as lipoprotein(a) and non-high-density cholesterol (non-HDL-c), as well.

Non-HDL-c has been recognized by the European Society of Cardiology and European Atherosclerosis Society, in their conjoint 2019 guidelines for the management of dyslipidemias, as one of the preferred lipoproteins to be measured in patients with type 2 diabetes mellitus or high triglyceride levels [2]. It is believed to provide an accurate estimate of total atherogenic particles concentration. The observed mismatch between low-density lipoprotein cholesterol (LDL-c) and non-HDL-c, occurring commonly in patients with type 2 diabetes [3] and metabolic syndrome [4], reinforces the importance of using non-HDL-c when evaluating patients, especially those at higher risk for ACVD.

Jan O. Aaseth et colleagues reported that non-HDL-c levels were considerably reduced in the Roux-en-Y gastric bypass (RYGB) group, in comparison to minor changes in the sleeve gastrectomy (SG) group. The authors concluded that RYGB was more effective than SG in the mitigation of dyslipidemia [1].

Despite the importance of this paper, we believe that the lack of information on antidyslipidemic drug treatment is an important limitation in the interpretation of its results. Depending on their ACVD risk, patients might be on very different treatment regimens before and after surgery. Considering drugs such as statins, ezetimibe, or fibrates, that impact LDL-c and triglycerides and, necessarily, non-HDL-c, is crucial to make inferences on lipid profile changes after bariatric surgery.

In our center, we evaluated 385 patients before and 12 months after bariatric surgery. After excluding all patients under treatment with statins, ezetimibe, and fibrates, we measured non-HDL-c changes in patients submitted to SG (*n* = 185) and RYGB (*n* = 18) (Table 1).

Whereas the investigators found mean non-HDL-c levels of 3.08 ± 0.77 mmol/L and 3.30 ± 0.92 mmol/L before RYGB and SG, respectively, we found higher mean non-HDL-c concentrations in both groups at baseline. After surgery, they reported a statistically significant 0.48 mmol/L non-HDL-c decrease (95% CI: 0.62; 0.33, *p* < 0.001) in patients submitted to RYGB whereas no statistically significant difference was found in patients submitted to SG [0.04 mmol/L (95% CI: −0.26;0.34, *p* = 0.789)].

In our sample, we identified statistically significant decreases in non-HDL-c after surgery in both groups, with bigger differences for RYGB than for SG. Compared to Jan O. Aaseth et colleagues who found a 15.6% and 1.21% non-HDL-c decrease in patients submitted to RYGB and SG, respectively, we found a 28.5% and 10.3% non-HDL-c decrease in patients submitted to the same respective procedures. Despite RYGB still demonstrating a higher effect according to these results, we would like to acknowledge the role of SG in reducing non-HDL-c. Our results propose a higher effect and statistical significance of the latter in non-HDL-c than those found by the authors. Many factors can explain these differences. Firstly, and most importantly, we performed this analysis after excluding drugs used to treat dyslipidemia, as previously mentioned. Secondly, the reporting of data collected in an interval between 6 and 12 months after surgery might have impacted the analysis of the results, since weight loss occurs predominantly in the first year, and patient status might vary considerably in that time interval. By contrast, our results reflect data collection 12 months after surgery. Thirdly, this paper included 21 patients submitted to SG, a sample size that could be underpowered to find changes in this group.

RYGB and SG are the commonest bariatric procedures performed worldwide [5]. SG is associated with less mal absorption and bone turnover issues than RYGB and thus is associated with fewer adverse effects [5], which might explain its popularity and growth in recent years. In patients with higher ACVD risk, RYGB could allow for better disease management, however, our results demonstrate that patients submitted to SG still benefit from a reduction in non-HDL-c.

## Figures and Tables

**Table 1:** Non-high-density lipoprotein cholesterol before and 12 months after bariatric surgery.

	*n*	Before Surgery	After Surgery	*p*	∆ (95% CI)
SG					
Non-HDL-c	185	3.79 ± 0.84	3.40 ± 0.85	<0.001	0.39 (0.29–0.49)
RYGB					
Non-HDL-c	18	3.89 ± 0.86	2.78 ± 0.76	<0.001	1.11 (0.68–1.54)

Legend—SG: sleeve gastrectomy; RYGB: Roux-en-Y gastric bypass; Non-HDL-c: non-high-density lipoprotein cholesterol; CI: Confidence interval.
